# Interactive Impact of Arbuscular Mycorrhizal Fungi and Elevated CO_2_ on Growth and Functional Food Value of *Thymus vulgare*

**DOI:** 10.3390/jof6030168

**Published:** 2020-09-09

**Authors:** Talaat H. Habeeb, Mohamed Abdel-Mawgoud, Ramy S. Yehia, Ahmed Mohamed Ali Khalil, Ahmed M. Saleh, Hamada AbdElgawad

**Affiliations:** 1 Biology Department, Faculty of Science at Yanbu, Taibah University, King Khalid Rd., Al Amoedi, Yanbu El-Bahr 46423, Saudi Arabia; thabeeb@yahoo.com; 2 Department of Medicinal and Aromatic Plants, Desert Research Centre, Cairo 11753, Egypt; Mohamed_drc@yahoo.com; 3 Department of Biological Sciences, College of Science, King Faisal University, Al-Ahsa 31982, Saudi Arabia; drramy4@hotmail.com; 4 Department of Botany and Microbiology, Faculty of Science, Cairo University, Giza 12613, Egypt; 5 Botany and Microbiology Department, Faculty of Science, Al-Azhar University, Cairo 13759, Egypt; khalilahmed_1980@hotmail.com; 6 Botany and Microbiology Department, Faculty of Science, Beni-Suef University, Beni-Suef 62511, Egypt

**Keywords:** mycorrhizae, elevated CO_2_, *Thymus vulgare*, growth, photosynthesis, metabolites, biological activity

## Abstract

Arbuscular mycorrhizal fungi (AMF) and elevated CO_2_ (eCO_2_) have been effectively integrated to the agricultural procedures as an ecofriendly approach to support the production and quality of plants. However, less attention has been given to the synchronous application of AMF and eCO_2_ and how that could affect the global plant metabolism. This study was conducted to investigate the effects of AMF and eCO_2_, individually or in combination, on growth, photosynthesis, metabolism and the functional food value of *Thymus vulgare*. Results revealed that both AMF and eCO_2_ treatments improved the photosynthesis and biomass production, however much more positive impact was obtained by their synchronous application. Moreover, the levels of the majority of the detected sugars, organic acids, amino acids, unsaturated fatty acids, volatile compounds, phenolic acids and flavonoids were further improved as a result of the synergistic action of AMF and eCO_2_, as compared to the individual treatments. Overall, this study clearly shows that co-application of AMF and eCO_2_ induces a synergistic biofertilization impact and enhances the functional food value of *T. vulgare* by affecting its global metabolism.

## 1. Introduction

Herbal plants have been widely used in traditional and folk medicine as an effectual solution to cure many diseases, being a big store for bioactive compounds, especially secondary metabolites [1]. They are known to have various biological activities such as antioxidant, antimicrobial, anti-inflammatory and anticancer properties [2]. Recently, a priority was given to herbal plants in terms of enhancing the production of the economically important phytochemicals through the application of cultivation procedures under stimulated growth conditions [3]. In this aspect, arbuscular mycorrhizal fungi (AMF) have been regarded as one of the most important beneficial microorganisms that are able to associate with almost two thirds of terrestrial plants improving their growth and stress tolerance [4]. In some cases, mycorrhizal symbiosis is essential as the host plant cannot grow normally and/or survive without it [5]. The beneficial effects of AMF symbiotic association with plants include enhanced levels of mineral nutrients and accumulation of primary and secondary metabolites [6]. From environmental point of view, AMF can keep the balance of soil aggregates, hence, able to fight erosion [7]. Accordingly, AMF represent a promising trend that has found its way in the sustainable agricultural productivity [8]. For instance, utilization of AMF for enhancing the production and quality of aromatic plants have been reported [8]. In this regard, several medicinal aromatic plants, such as pennyroyal and parsley, showed enhanced levels of bioactive metabolites when associated with AMF [6].

In another aspect, the exposure of plants to elevated CO_2_ (eCO_2_) has been regarded as an effective approach to improve the nutritional and medicinal values of herbal plants [9]. eCO_2_ can increase plant growth and productivity either directly by enhancing photosynthesis [10] or indirectly by stimulating plant water use efficiency [11]. On the other hand, the effect of eCO_2_ on belowground communities, including AMF, are still not fully understood [12]. What is well known is that the higher the photosynthetic activity, under eCO_2_, the more the photosynthate transfer to plant roots and the higher release to the associated microbial communities [13]. Furthermore, as being dependent on their host plant for carbon, AMF may be sensitive to global climatic changes that influence their host [14]. Therefore, such triple effect resulting from interactions among plant, AMF and eCO_2_ is expected to have beneficial roles in increasing the productivity and quality of crops and medicinal plants.

One of the well-known plants for both culinary and medicinal purposes is *Thymus vulgaris* L., a member of the family Lamiaceae, being widely used in folk medicine for treatment of several diseases like bedwetting, diarrhea, stomach ache, arthritis, sore throat, cough, bronchitis and chest congestion [15]. The biological activities of *T. vulgaris* are mainly ascribed to its content of secondary metabolites, particularly essential oils that have been extensively studied for antioxidant, antimicrobial and antitumor activities [16]. Thus, improving the accumulation of these phytochemicals in *T. vulgaris* could support its nutritional, medicinal and pharmacological properties. In this regard, previous studies have reported the positive impacts of both AMF and eCO_2_ on the growth and quality of herbal plant [17,18], however, the complete picture on how AMF-eCO_2_ combination affect primary and secondary metabolomes is not fully drawn [19,20]. In addition, metabolic profiling of the host plant is essential to understand the mechanisms behind the changes occurring in response to the individual and/or combined effect of AMF and eCO_2_. So far, the detailed metabolic implications induced by the synchronous application of eCO_2_ and AMF on plants are not investigated. Thus, the current study was conducted to explore, in details, the individual and combined impacts of eCO_2_ and AMF on *T. vulgaris*, as a model herbal plant. We have assessed the changes in mycorrhizal colonization, plant biomass production, photosynthesis, respiration and levels of individual primary (sugars, amino acids, fatty acids and organic acids), secondary (phenolic acids and flavonoids) metabolites and volatile oils. Further, the associated changes in nutritional and medicinal values of *T. vulgaris* were investigated.

## 2. Material and Method

### 2.1. Experimental Setup, Plant Materials and Growth Conditions

Soil potting was mixed with sterilized sand (1:3) and inculcated with a pure commercial inoculum of *Rhizophagus irregularis* (MUCL 41,833 obtained from Glomeromycota in vitro collection (GINCO)) at a concentration of 50 spores per soil in a pot (25 × 15 cm). The control treatments were represented by non-inoculated soil. The seeds of *T. vulgaris* were disinfected then sown in both treated and non-treated soils. Plants were grown in a controlled greenhouse at 21/18 °C, 16/8 h day/night, and 60% humidity, they were regularly watered. The pots inn each of the control and AMF-inoculated groups were equally subdivided into two sub-groups, one subjected to 410 ppm CO_2_ (ambient CO_2_; aCO_2_) and the other subjected to 620 ppm CO_2_ (elevated CO_2_; eCO_2_,) through the time course of the experiment. The plants were harvested after 6 weeks, then the aerial parts were immediately frozen in liquid nitrogen and stored at −20 °C to be used in different plant analyses. For determination of dry matter and mineral elements, plant shoots were washed with distilled water and dried at 75 °C for 72 h.

### 2.2. Mycorrhizal Parameters

Mycorrhizal colonization was demonstrated following Phillips and Hayman [21]. About 0.5 g of fresh roots were clarified with potassium hydroxide (10% *w*/*v*) and potassium hydroxide (10%) + hydrogen peroxide (10% *v*/*v*) in a ratio of 1:1 (*v*/*v*), then stained with 0.05% trypan blue in lactoglycerol. A stereomicroscope (40×) was used to show the stained roots, while the colonization rate was calculated by using gridline intersect method [22].

### 2.3. Photosynthesis Parameters

The light-saturated photosynthetic rates (μmol CO_2_ m^−2^ s^−1^) of mature leaves were measured (LI-COR LI- 6400, LI-COR Inc., Lincoln, NE, USA), according to AbdElgawad et al. [23]. Dark respiration was determined as the absolute CO_2_ exchange rate determined at photosynthetic photon flux density (μmolm^−2^ s^−1^).

### 2.4. Metabolic Profiling

For extraction of sugars, plant tissues were homogenized in 50% (*v*/*v*) acetonitrile. The method described by Hamad et al. [24] was applied to identify the individual sugars in the plant extract by using high-performance liquid chromatography (HPLC), then comparing their retention time with those of a standard mixture. Quantification of the sugar samples was achieved based on peak area comparison with a calibration curve of the corresponding standards. Organic acids were extracted in phosphoric acid (0.1% *v*/*v*) supplemented with butylated hydroxyanisole (3 g/L) and then analyzed using HPLC with a SUPELCOGEL C-610H column coupled to a UV detection system set at 210 nm (LaChromL-7455 diode array, LaChrom, Tokyo, Japan). The concentration of each organic acid was calculated by using a calibration curve [24]. For extraction of amino acids, a known weight of plant tissues was vigorously homogenized in 80% aqueous ethanol. Amino acids were measured using a Waters Acquity UPLC-tqd system (Milford, Worcester County, MA, USA) equipped with a BEH amide 2.1 × 50 column. The lipophilic fraction of plant samples was obtained by extraction in chloroform/methanol (2:1, *v*/*v*). Thereafter, fatty acids were detected, according to Hassan et al. [25], by using GC/MS analysis (Hewlett Packard, Palo Alto, CA, USA) with an HP-5 MS column (30 m × 0.25 mm × 0.25 mm). Fatty acids were quantified using NIST 05 database and Golm ** Metabolome Database (http://gmd.mpimp-golm.mpg.de). Phenolic acids and flavonoids were extracted in acetone-water solution (4:1 *v*/*v*) for 24 h. The method outlined in Hamad et al. [24] was followed up for determination of Phenolic acids and flavonoids using an HPLC system (SCL-10A vp, Shimadzu Corporation, Kyoto, Japan). The concentration of each compound was calculated with a calibration curve of the corresponding standard. For extraction of volatile oils, two hundred gm of fresh plant material were subjected to steam distillation with about 500 mL of water, where the volatiles were collected [26]. The levels of volatiles were determined using gas chromatography–mass spectrometry (GC–MS) according to the method outlined by El Hattab et al. [27].

### 2.5. Determination of Biological Activities

Several methods were used to determine the total antioxidant capacities of the plant extract, including the ferric reducing antioxidant power (FRAP), oxygen radical absorbance capacity (ORAC), inhibition of LDL (low density lipoprotein) oxidation (TBARS and conjugated dienes) and inhibition of hemolysis assays [23,24]. For LDL oxidation, dialyzed LDL (100 μg protein/mL) was diluted in 10 mM PBS (phosphate buffered saline containing 0.01 Mphosphate-buffer and 0.15 M NaCl, pH 7.4) and incubated at 37 °C in presence or absence of 10 μM CuSO_4_. Oxidation was performed with or without the sample solution of colostrum proteins. After incubation, lipid peroxidation of the LDL was measured. Thiobarbituric acid reactive substances (TBARS) was determined at 532 nm/600 nm, using 1,1,3,3-Tetramethoxypropane as standard for calibration curve, while conjugated diene formation was measured at 232 nm of LDL solution (100 μg protein/mL) in PBS incubated with CuSO_4_ (10 μM) in the absence or presence of various concentrations of bovine colostrums protein [28].

### 2.6. Statistical Analysis

Experiments were carried out following a randomized complete block design. Data normality and the homogeneity of variances were checked using the Kolmogorove–Smirnov test and Levene’s test, respectively. Each experiment was done in five replicates (n = 5). All the data was subjected to one-way analysis of variance (ANOVA). Student’s *t*-test at probability levels of 0.05, 0.01 or 0.001 was used to test the difference between the treatment and control or between AMF alone and the combined AMF+eCO_2_ treatment. All statistical tests were performed using the computer program PASW statistics 18.0 (SPSS Inc., Chicago, IL, USA).

## 3. Results and Discussion

### 3.1. AMF Colonization and Hyphal Growth

It is known that AMF are largely dependent on their host plant for carbon, so they are sensitive to climatic changes that affect their host plant [14]. In this sense, eCO_2_ could have an indirect effect on mycorrhizal colonization by promoting carbon assimilation and allocation to roots [29]. Since AMF are attached to plant roots, they are lucky to receive higher amount of photosynthates under eCO_2_ before other soil microbes [30]. Herein, the mycorrhizal growth was significantly enhanced in *T. vulgare* by AMF treatments (Table 1). Such mycorrhizal proliferation was much more stimulated under eCO_2_ conditions. Several studies have demonstrated some positive effects for eCO_2_ on AMF-plant association such as increased mycorrhizal root length [14] and increased extra-radical hyphae [31]. However, other studies did not show any beneficial effects for eCO_2_ on AMF growth in host plants [32,33]. Therefore, the impact of eCO_2_ levels on mycorrhizal growth seems to be dependent on plant species, AMF species and soil type [34]. In fact, as being a member of *Glomeraceae*, the ratio of *R. irregularis* has been reported to be more positively influenced by eCO_2_ than others, e.g., *Gigasporaceae* [35].

### 3.2. AMF and eCO_2_ Acts Synergistically to Improve Photosynthetic Capacity and Biomass Production

**Figure 1:**
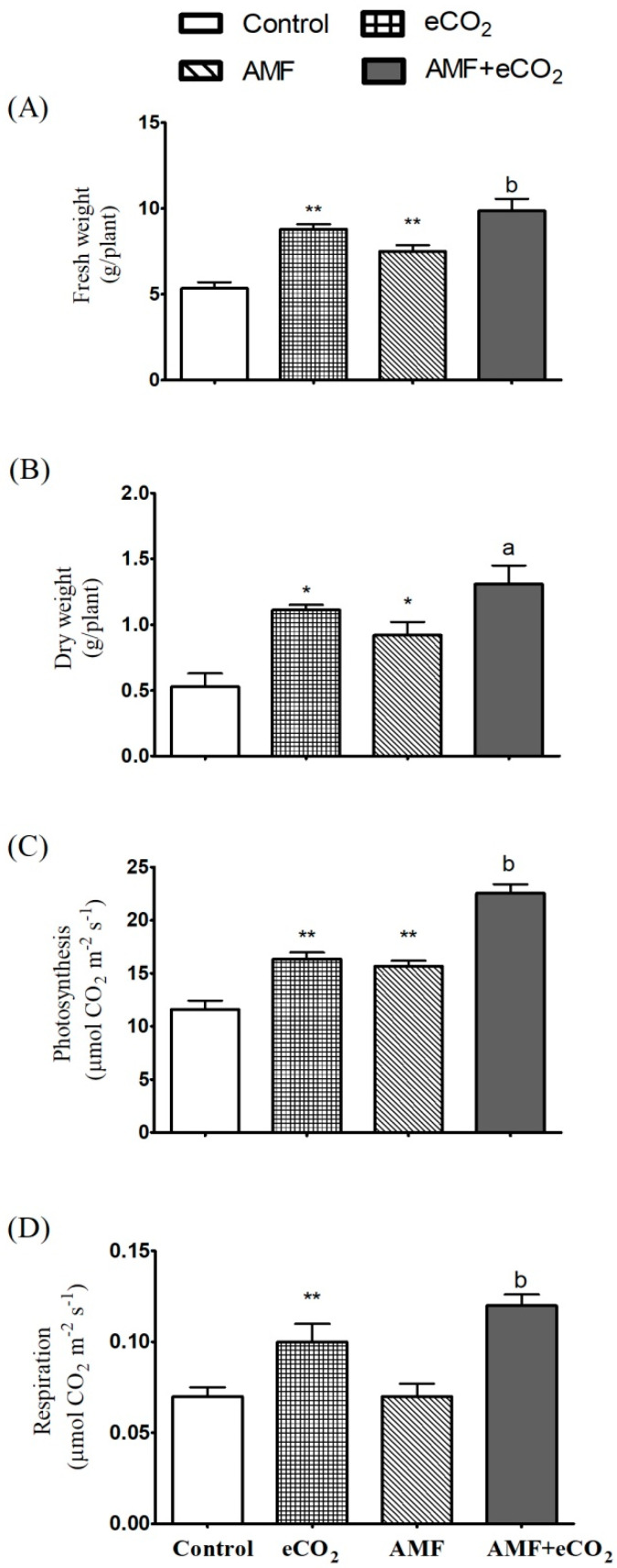
Fresh mass (**A**), dry mass (**B**), and rates of photosynthesis (**C**) and respiration (**D**) in *Thymus vulgare* grown under normal conditions (control) or the effect of eCO_2_ (620 ppm), AMF or their combination (AMF-eCO_2_). Values are mean ± standard error of five independent replicates. Asterisks indicate significant changes (* *p* < 0.05; ** *p* < 0.01) compared to control, as revealed by the Student’s *t*-test. Lowercase letters indicate significant differences (^a^ *p* < 0.05; ^b^ *p* < 0.01) between AMF alone and the combined AMF+eCO_2_ treatment.

It has been known that the photosynthetic rate, and consequently biomass production, could be improved under the effect of AMF inoculation, as a result of the expected increased nutrients uptake [36], and also under eCO_2_ atmosphere due to the enhancement of the carboxylation reaction of rubisco [37]. Supporting this hypothesis, the current results revealed that eCO_2_ and AMF independently, and to more extent in combination, promoted photosynthetic rate and biomass production in *T. vulgare* (Figure 1). Such increments were much more induced by the interaction between both treatments.

Similar to our results, the positive effects of eCO_2_ on biomass of *T. vulgaris* and some other medicinal plants, *Ocimum basilicum*, *Origanum vulgare*, *Mentha piperita* and *Mentha spicata*, have been previously investigated [38]. Moreover, the increased biomass production in plants inoculated with AMF was reported [39]. Regarding the interaction between eCO_2_ and AMF, It is well known that eCO_2_ stimulates the photosynthetic rate and plant growth [40], which in turn, affects the allocation of photosynthates to AMF, consequently makes more C available to AMF colonizing the roots [13], thus, increasing sink strength in mycorrhizal plants, and eventually this leads to increased C storage in soils [41]. Such effect is hypothesized to create a balance between carbon cost and nutrient benefits, besides reducing the negative effects of down regulation of photosynthesis caused by acclimation of plants to long-term exposure to eCO_2_ [42]. In this regard, it has been found that both eCO_2_ and AMF, when applied individually or in combination, improved biomass production of *Pisum sativum* and lettuce [32,43]. In addition, it has been indicated that mycorrhizal plants have higher photosynthetic rate [33] when grown under high CO_2_ levels. However, such an effect might differ among variable cultivars [44]. In contrast, it was supposed that eCO_2_ may impair the beneficial effects of AMF on plant biomass, especially when the fungal community is dominated by *Glomus* species [45]. This might be due to the difference among AMF taxa in their exchange of carbon and nutrients [46].

### 3.3. Application of AMF and eCO_2_ Improves the Nutritional Value of T. vulgare

**Figure 2:**
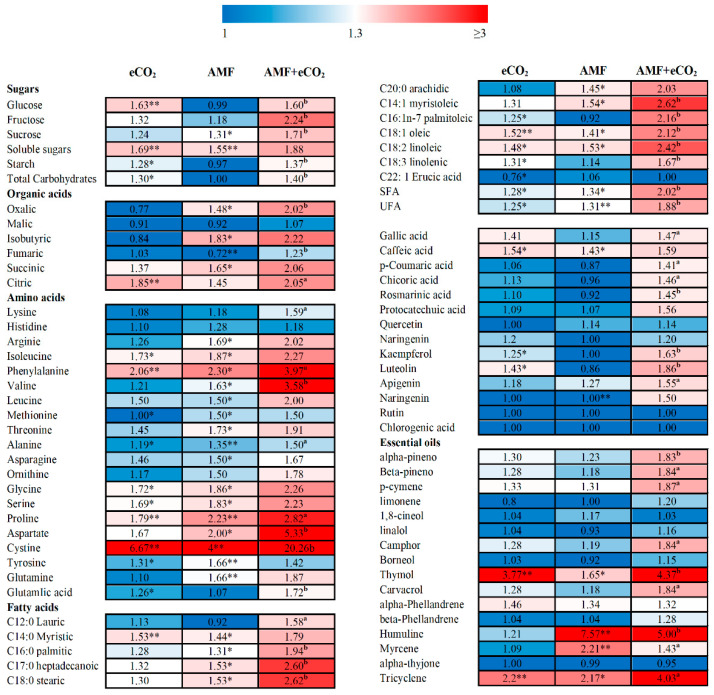
Heatmap of fold change in the contents of primary and secondary metabolites of *Thymus vulgare* grown under the effect of eCO_2_ (620 ppm), AMF or their combination (AMF-eCO_2_). Asterisks indicate significant (* *p* < 0.05; ** *p* < 0.01) increased fold changes compared to control (untreated plants), as revealed by Student’s t-test. Lowercase letters indicate significant differences (^a^ *p* < 0.05; ^b^ *p* < 0.01) between AMF alone and the combined AMF+eCO_2_ treatment.

It was assumed that the nutritive value of plants is highly related to its content of primary metabolites, e.g., sugars, proteins and lipids [9]. In this regard, sugars and organic acids are related to taste and flavor [47], essential amino acids are involved in some biological processes, such as protein synthesis [48] and a lower saturated/unsaturated fatty acids (SFA/USFA) ratio is linked to cardio-protective effects [49]. It has been reported that the higher the CO2 levels, the higher the rate of photosynthetic activity, which is linked to the enhancement of the carboxylation reaction of rubisco, the enzyme responsible for CO_2_ fixation [37]. As a consequence of photosynthesis improvement, sugars could be accumulated and also broken down via dark respiration, resulting in production of the precursors necessary for synthesis of different classes of primary and secondary metabolites [50]. Supporting such a concept, the individual AMF and eCO_2_ treatments induced significant increases in the content of total soluble sugar of *T. vulgare,* about 1.6 folds, however, starch was significantly accumulated under eCO_2_ only (Table 2, Figure 2). Further, the synchronous application of AMF and eCO_2_ caused a significant accumulation in the levels of the majority of the measured sugars relative to AMF alone treatment. Similarly, CO_2_ enrichment enhanced the accumulation of sucrose and starch in oil palm [51], and increased the accumulation of total soluble carbohydrates and starch in ginger varieties [52]. AMF treatments were found to induce the accumulation of total soluble sugars in lettuce [53]. Further, the interaction between AMF and CO_2_ improved forage quality of alfalfa plants by increasing the levels of glucose, fructose and hemicellulose and decreasing that of lignin [54].

Besides, the current results revealed that the combined AMF and eCO_2_ treatment induced a significant increase in the majority of the detected organic acids, amino acids (including both essential and non-essential amino acids) and fatty acids in *T. vulgare*, relative to AMF alone (Table 2, Figure 2). Regarding the individual treatments, AMF was more efficient in inducing the accumulation of these primary metabolites than eCO_2_. All AMF and/or eCO_2_ treatments did not affect the SFA/USFA ratio. Similarly, it was reported that AMF-inoculated maize plants, under low temperature, had higher amino acid concentrations than non-mycorrizal ones, especially for Thr, Lys, Gly, Ala and His contents [55]. In contrast, proline content was reduced in mycorhizal *Capsicum annuum* grown under saline conditions [56]. Moreover, different effects of eCO_2_ on amino acids were reported, which were reduced in barley [57], increased in spring wheat [58], or were not affected in maize [59]. It was also shown that organic acid levels were increased in mycorrhizal *Pinus sylvestris* grown under heavy metal concentrations [60], while they were not increased in *Portulacaria afra* under eCO_2_ [61]. The concentration of most fatty acids of soybean was unchanged under higher levels of CO_2_ [62]. On the other hand, a significant increase in the levels of individual fatty acids was reported in parsley and dill grown under eCO_2_, which is more evident on UFA than SFA [9]. Therefore, the synchronous application of AMF and eCO_2_ could be beneficial to avoid the negative impact of the individual treatment and/or to support their positive effects.

### 3.4. AMF and eCO_2_ Promote the Accumulation of Phenolic Compounds and Volatile Oils in T. vulgare

Mycorrhizal symbiosis with medicinal plants has been recognized to induce the accumulation of secondary metabolites, especially phenolic compounds which play an important role in curing several ailments [63]. The present results showed that protocatechuic, p-coumaric and rosmarinic acids are the most abundant phenolic acids; while apigenin, kaempferol, quercetin and luteolin are the predominant flavonoid in *T. vulgare* (Table 3). Similarly, previous studies revealed the presence of some phenolic acids such as cinnamic, carnosic and rosmarinic acids, and also flavonoids such as luteolin and apigenin derivatives in *T. vulgare* [64]. There was a significant increment in the levels of the majority of the detected phenolic acids and flavonoids in *T. vulgare* under AMF and/or eCO_2_ treatments, however the combined treatment was much more efficient than the individual ones (Figure 2). On the other hand, in consistence with the previous studies [65], the present results revealed the presence of 16 volatile oils in *T. vulgare*, whereas 1,8-cineol, carvacrol and p-cymene are the most dominant followed by less amounts of linalol, α- and β-pineno, α-Phellandrene, myrecene and thymol (Table 3). There is also a significant increase in the volatile oils of *T. vulgare*, under the individual and combined treatments.

Supporting our results, several studies have investigated the potential effects of eCO_2_ and AMF, separately and in combination, on the levels of phenolic compounds and antioxidant activity in a variety of plant species. For instance, AMF treatments caused an increase in phenolic compounds content of lettuce [53,66], and in the antioxidant capacity of sweet basil [67]. Moreover, the flavonoids of some wild plants, such as *Libidibia ferrea*, were found to be accumulated by mycorrhizal association [68]. Similarly, eCO_2_ induced the accumulation of some phenolic compounds in birch [69], and *Zingiber officinale* [52]. However, a low phenolic content was reported for some plants such as rice [70] under eCO_2_. Regarding the interaction between eCO_2_ and AMF, it was reported that the induction of secondary metabolites in lettuce and alfalfa by AMF was negatively affected under eCO_2_, probably due to utilization of photoassimilates for increasing plant biomass and for AMF growth as well, at the expense of secondary metabolism [43,44]. Therefore, it could be suggested that climatic changes might have an impact on AMF, which in turn, affect the metabolic functions of their host plants.

Several scenarios have been proposed to explain the induction of secondary metabolites in response to AMF associations and eCO_2_. It was found that AMF could affect secondary metabolites through improved photosynthesis and mineral content of the host plants, activation of pathways involved in synthesis of secondary metabolites, or higher expression of some genes related to secondary metabolism [68]. On the other hand, the eCO_2_-induced changes in plant secondary metabolism have been attributed to either excess amount of non-structural carbohydrates, resulting in an increment in carbon-based secondary metabolites [9,37,51].

### 3.5. AMF and eCO_2_-Induced Changes in Secondary Metabolites Support the Biological Activities of T. vulgare

Reactive oxygen species and free radicals have been recognized to induce harmful effects on living organisms. In this aspect, antioxidants, such as phenolic compounds and volatile oils, could act as free radical scavengers [71]. The present results showed an increase in the total antioxidant capacities of *T. vulgare*, tested by different methods (FRAP, ORAC, inhibition of LDL oxidation (TBARS and conjugated dienes) and inhibition of hemolysis), under the effects of AMF and/or eCO_2_ (Table 3). It was previously reported that the antioxidant properties exhibited by *T. vulgare* extracts have been attributed to their content of volatile oils, especially carvacrol and thymol [72], flavonoids such as apigenin and luteolin derivatives and phenolic acids such as cinnamic and rosmarinic acids [64]. Moreover, some phenolic compounds were previously isolated from *T. vulgare* and proved to inhibit oxidative hemolysis [73]. The decreased levels of lipid peroxidation products such as TBARS and conjugated dienes might be ascribed to some protective effects of thymol [74].

## 4. Conclusions

Based on the above results, it is clear that the tested plant, *T. vulgare*, has benefited from the independent and combined effects of both AMF and eCO_2_, however their synchronous application is much more beneficial. Such positive impacts are being reflected on improved biomass production and higher accumulation of primary (sugars, amino acids, fatty acids and organic acids), and secondary (phenolic acids, flavonoids and volatile oils) metabolites. *T. vulgare* plants grown under synchronous application of AMF and eCO_2_ have taken much advantage over those grown under the individual effects of both factors in terms of improved growth and bioactive components. Thus, the current study clearly shows that co-application of AMF and eCO_2_ is a promising approach to improve the growth and the nutritional and health promoting values of *T. vulgare*. Further, the robust monitoring of primary and secondary metabolites presented herein could support our understanding about the mechanisms behind the positive impacts of AMF and eCO_2_ on plants.

## Figures and Tables

**Table 1:** Mycorrhizal colonization and growth parameters in roots of *Thymus vulgare* grown under normal conditions (control) or the effect of eCO_2_ (620 ppm), arbuscular mycorrhizal fungi (AMF) or their combination (AMF-eCO_2_). Values are mean ± standard error of five independent replicates. Asterisks indicate significant changes (*** *p* < 0.001) between AMF alone and the combined AMF+eCO_2_ treatment.

Metabolite	Control	eCO_2_	AMF	AMF + eCO_2_
Colonization (% root)	nd	nd	33.06 ± 2.39	54.04 ± 1.11 ***
Hyphal length (cm g^−1^ soil)	nd	nd	12.94 ± 5.82	19.10 ± 9.74 b ***
Number of arbuscules (no. cm^−1^ root)	nd	nd	4.78±0.27	5.03 ± 0.18

nd = not detected.

**Table 2:** Levels of primary metabolites (mg g^−1^ dry weight) in *Thymus vulgare* grown under normal conditions (control) or the effect of eCO_2_ (620 ppm), AMF or their combination (AMF-eCO_2_). Values are mean ± standard error of five independent replicates. Asterisks indicate significant changes (* *p* < 0.05; ** *p* < 0.01) compared to control, as revealed by the Student’s t-test. Lowercase letters indicate significant differences (^a^ *p* < 0.05; ^b^ *p* < 0.01) between AMF alone and the combined AMF+eCO_2_ treatment.

	Control	eCO_2_	AMF	AMF + eCO_2_
**Sugars**				
Glucose	1.34 ± 0.07	2.18 ± 0.12 **	1.32 ± 0.07	2.14 ± 0.01 ^b^
Fructose	0.34 ± 0.05	0.45 ± 0.02	0.4 ± 0.02	0.76 ± 0.06 ^b^
Sucrose	1.67 ± 0.15	2.07 ± 0.15	2.19 ± 0.04 *	2.85 ± 0.1 ^b^
Soluble sugars	6.09 ± 0.31	10.29 ± 0.3 **	9.46 ± 0.35 **	11.44 ± 0.8
Starch	62.06 ± 1.89	79.26 ± 4.63 *	60.1 ± 1.7	85.22 ± 1.68 ^b^
Total carbohydrates	105.38 ± 3.08	137.1 ± 6.75 *	105.62 ± 6.31	147.79 ± 2.01 ^b^
**Organic acids**				
Oxalic	3.84 ± 0.33	2.97 ± 0.06	5.7 ± 0.34 *	7.75 ± 0.29 ^b^
Malic	6.88 ± 0.3	6.25 ± 0.34	6.36 ± 0.29	7.38 ± 0.36
Isobutyric	3.46 ± 0.33	2.89 ± 0.46	6.34 ± 0.67 *	7.67 ± 0.53
Fumaric	0.93 ± 0.01	0.96 ± 0.08	0.67 ± 0.04 **	1.14 ± 0.07 ^b^
Succinic	3.07 ± 0.33	4.21 ± 0.29	5.08 ± 0.39 *	6.31 ± 0.58
Citric	2.88 ± 0.33	5.32 ± 0.28 **	4.17 ± 0.54	5.9 ± 0.2 ^a^
**Essential amino acids (EAAs)**				
Histidine	2.49 ± 0.19	2.75 ± 0.3	3.19 ± 0.23	2.95 ± 0.38
Isoleucine	0.15 ± 0.02	0.26 ± 0.03 *	0.28 ± 0.03 *	0.34 ± 0.01
Leucine	0.02 ± 0	0.03 ± 0	0.03 ± 0 *	0.04 ± 0
Lysine	4.2 ± 0.21	4.55 ± 0.29	4.97 ± 0.25	6.67 ± 0.5 ^a^
Methionine	0.02 ± 0	0.02 ± 0 *	0.03 ± 0 *	0.03 ± 0
Phenylalanine	0.33 ± 0.04	0.68 ± 0.03 **	0.76 ± 0.1 *	1.31 ± 0.08 ^a^
Valine	0.48 ± 0.05	0.58 ± 0.06	0.78 ± 0.08 *	1.72 ± 0.07 ^b^
Threonine	0.11 ± 0.01	0.16 ± 0.02	0.19 ± 0.02 *	0.21 ± 0.01
Arginine	1.86 ± 0.13	2.35 ± 0.25	3.14 ± 0.34 *	3.76 ± 0.17
Total EAAs	9.66	11.38	13.37	17.03
**Non-essential amino acids (NEAAs)**				
Aspartate	0.03 ± 0	0.05 ± 0	0.06 ± 0.01 *	1.51 ± 0.1 ^b^
Cystine	0.03 ± 0	0.2 ± 0.02 **	0.12 ± 0.01 **	0.62 ± 0.06 ^b^
GlutamIic acid	77.02 ± 5.21	97.05 ± 3.71 *	82.21 ± 5.27	132.44 ± 6.78 ^b^
Glutamine	96.66 ± 6.05	106.65 ± 0.98	159.99 ± 6.47 **	180.39 ± 9.12
Asparagine	1.23 ± 0.12	1.8 ± 0.19	1.84 ± 0.13 *	2.05 ± 0.09
Glycine	1.18 ± 0.13	2.03 ± 0.22 *	2.2 ± 0.24 *	2.67 ± 0.29
Ornithine	0.18 ± 0.03	0.21 ± 0.02	0.27 ± 0.04	0.32 ± 0.02
Proline	1.25 ± 0.06	2.24 ± 0.08 **	2.79 ± 0.15 **	3.52 ± 0.19 ^a^
Serine	0.35 ± 0.04	0.59 ± 0.06 *	0.64 ± 0.07 *	0.78 ± 0.08
Tyrosine	0.99 ± 0.11	1.3 ± 0.02 *	1.64 ± 0.06 **	1.41 ± 0.09
Alanine	18.65 ± 0.97	22.22 ± 0.76 *	25.09 ± 0.67 **	28.06 ± 0.53 ^a^
Total NEAAs	197.57	234.34	276.85	353.77
**Saturated fatty acids (SFA)**				
Lauric (C12:0)	1.42 ± 0.17	1.6 ± 0.15	1.31 ± 0.26	2.24 ± 0.19 ^a^
Tetradecanoic (C14:0)	1.7 ± 0.15	2.6 ± 0.12 **	2.44 ± 0.14 *	3.05 ± 0.18
Hexadecanoic (C16:0)	10.18 ± 0.87	13.08 ± 0.73	13.38 ± 0.55 *	19.8 ± 1.14 ^b^
Heptadecanoic (C17:0)	0.62 ± 0.06	0.81 ± 0.09	0.95 ± 0.09 *	1.62 ± 0.09 ^b^
Octadecanoic (C18:0)	2.26 ± 0.21	2.96 ± 0.33	3.46 ± 0.34 *	5.9 ± 0.33 ^b^
Eicosanoic (C20:0)	2 ± 0.19	2.16 ± 0.19	2.9 ± 0.14 *	4.06 ± 0.4
Total SFA	18.18 ± 1.15	23.2 ± 0.68 *	24.43 ± 0.94 *	36.68 ± 0.38 ^b^
**Unsaturated fatty acids**				
Myristoleic (C14:1)	0.61 ± 0.06	0.8 ± 0.09	0.94 ± 0.09 *	1.6 ± 0.09 ^b^
Palmitoleic (C16:1n-7)	1.61 ± 0.08	2.02 ± 0.1 *	1.48 ± 0.16	3.47 ± 0.19 ^b^
Octadecenoic (C18:1)	7.45 ± 0.7	11.29 ± 0.35 **	10.51 ± 0.24 *	15.82 ± 0.23 ^b^
Erucic acid (C22: 1)	12.19 ± 0.73	9.22 ± 0.28 *	12.93 ± 2.31	12.2 ± 0.54
Octadecadienoic (C18:2)	16.68 ± 1.57	24.65 ± 2.14 *	25.5 ± 1.48*	40.43 ± 0.78 ^b^
Octadecatrienoic (C18:3)	5.36 ± 0.5	7.01 ± 0.28 *	6.1 ± 0.28	8.97 ± 0.28 ^b^
USFA	43.9 ± 2.2	55 ± 2.55 *	57.46 ± 0.63 **	82.48 ± 0.68 ^b^
**SFA/USFA**	0.35	0.34	0.35	0.35

**Table 3:** Levels of phenolic compounds and volatile oils (mg g^−1^ dry weight) and biological activities in *Thymus vulgare* grown under normal conditions (control) or the effect of eCO_2_ (620 ppm), AMF or their combination (AMF-eCO_2_). Values are mean ± standard error of five independent replicates. Asterisks indicate significant changes (* *p* < 0.05; ** *p* < 0.01) compared to control, as revealed by Student’s t-test. Lowercase letters indicate significant differences (^a^ *p* < 0.05; ^b^ *p* < 0.01) between AMF alone and the combined AMF+eCO_2_ treatment.

Metabolite	Control	eCO_2_	AMF	AMF + eCO_2_
**Phenolic acids**				
Caffeic acid	0.46 ± 0.04	0.71 ± 0.04 *	0.66 ± 0.03 *	0.73 ± 0.03
Chlorogenic acid	0.01 ± 0	0.01 ± 0	0.01 ± 0	0.01 ± 0 ^a^
Protocatechuic acid	4.11 ± 0.32	4.47 ± 0.35	4.38 ± 0.45	6.42 ± 0.6
Gallic acid	0.34 ± 0.02	0.48 ± 0.06	0.39 ± 0.03	0.5 ± 0.02 ^a^
p-Coumaric acid	2.66 ± 0.22	2.82 ± 0.35	2.32 ± 0.26	3.76 ± 0.36 ^a^
Chicoric acid	1.28 ± 0.13	1.44 ± 0.15	1.23 ± 0.15	1.87 ± 0.16 ^a^
Rosmarinic acid	1.72 ± 0.2	1.89 ± 0.1	1.59 ± 0.1	2.5 ± 0.11 ^b^
**Flavonoids**				
Quercetin	0.07 ± 0	0.07 ± 0	0.08 ± 0.01	0.08 ± 0.01
Naringenin	0.05 ± 0	0.06 ± 0	0.05 ± 0.01	0.06 ± 0.01
Kaempferol	0.08 ± 0	0.1 ± 0.01 *	0.08 ± 0	0.13 ± 0.01 ^b^
Luteolin	0.07 ± 0	0.1 ± 0.01 *	0.06 ± 0	0.13 ± 0.01 ^b^
Apigenin	0.11 ± 0.02	0.13 ± 0	0.14 ± 0.01	0.17 ± 0.01 ^a^
Rutin	0.01 ± 0	0.01 ± 0	0.01 ± 0	0.01 ± 0
**Volatile oils**				
alpha-pineno	1.62 ± 0.21	2.1 ± 0.08	2 ± 0.03	2.97 ± 0.1 ^a,b^
Beta-pineno	2.79 ± 0.36	3.57 ± 0.27	3.29 ± 0.43	5.13 ± 0.23 ^a^
p-cymene	4.45 ± 0.32	5.94 ± 0.48	5.85 ± 0.51	8.31 ± 0.37 ^a^
limonene	0.1 ± 0.03	0.08 ± 0.01	0.1 ± 0	0.12 ± 0.02
1,8-cineol	8.23 ± 1.04	8.54 ± 1.19	9.61 ± 1.34	8.51 ± 0.73
linalol	3.42 ± 0.44	3.54 ± 0.49	3.19 ± 0.7	3.96 ± 0.23
Camphor	0.64 ± 0.08	0.82 ± 0.09	0.76 ± 0.1	1.18 ± 0.09 ^a^
Borneol	0.39 ± 0.05	0.4 ± 0.06	0.36 ± 0.08	0.45 ± 0.09
Thymol	1.5 ± 0.19	5.66 ± 0.48 **	2.47 ± 0.28 *	6.55 ± 0.48 ^b^
Carvacrol	4.97 ± 0.63	6.35 ± 0.68	5.85 ± 0.76	9.13 ± 0.15 ^a^
alpha-Phellandrene	2.16 ± 0.21	3.15 ± 0.34	2.9 ± 0.38	2.86 ± 0.17
beta-Phellandrene	0.25 ± 0.03	0.26 ± 0.03	0.26 ± 0.04	0.32 ± 0.04
Humuline	0.14 ± 0.01	0.17 ± 0.01	1.06 ± 0.05 **	0.7 ± 0.04 ^b^
Myrcene	1.84 ± 0.12	2 ± 0.4	4.07 ± 0.41 **	2.64 ± 0.12 ^a^
alpha-thyjone	1.1 ± 0.05	1.1 ± 0.04	1.09 ± 0.08	1.04 ± 0.03
Tricyclene	0.3 ± 0.02	0.66 ± 0.04 **	0.65 ± 0.07 *	1.21 ± 0.14 ^a^
**Antioxidant capacity (FRAP)**	17.08 ± 1.8	27.57 ± 1 **	22.85 ± 1.13	35.91 ± 0.91 ^b^
**Oxygen radical absorbance capacity (ORAC)**	743.41 ± 33.19	1034.6 ± 108.25	983.99 ± 35.17 **	1680.57 ± 81.71 b
**% inhibition of LDL oxidation**				
TBARS)	14.08 ± 0.99	26.56 ± 0.76 **	24.55 ± 0.98 **	35.32 ± 2.25 ^a^
conjugated dienes	17.7 ± 2.1	32.56 ± 0.51 **	28.51 ± 1.16 *	43.53 ± 1.29 ^b^
**% inhibition of hemolysis**	13.4 ± 0.83	22.02 ± 1.97 *	16.45 ± 1.05	28.28 ± 1.08 ^b^
